# Innate Immune Recognition: Implications for the Interaction of *Francisella tularensis* with the Host Immune System

**DOI:** 10.3389/fcimb.2017.00446

**Published:** 2017-10-16

**Authors:** Zuzana Krocova, Ales Macela, Klara Kubelkova

**Affiliations:** Department of Molecular Pathology and Biology, Faculty of Military Health Sciences, University of Defence, Hradec Kralove, Czechia

**Keywords:** innate immunity, intracellular bacteria, immune recognition, *Francisella tularensis*, signaling windows concept, spatiotemporal network

## Abstract

The intracellular bacterial pathogen *Francisella tularensis* causes serious infectious disease in humans and animals. Moreover, *F. tularensis*, a highly infectious pathogen, poses a major concern for the public as a bacterium classified under Category A of bioterrorism agents. Unfortunately, research has so far failed to develop effective vaccines, due in part to the fact that the pathogenesis of intracellular bacteria is not fully understood and in part to gaps in our understanding of innate immune recognition processes leading to the induction of adaptive immune response. Recent evidence supports the concept that immune response to external stimuli in the form of bacteria is guided by the primary interaction of the bacterium with the host cell. Based on data from different *Francisella* models, we present here the basic paradigms of the emerging innate immune recognition concept. According to this concept, the type of cell and its receptor(s) that initially interact with the target constitute the first signaling window; the signals produced in the course of primary interaction of the target with a reacting cell act in a paracrine manner; and the innate immune recognition process as a whole consists in a series of signaling windows modulating adaptive immune response. Finally, the host, in the strict sense, is the interacting cell.

## Introduction

The mammalian immune system defends against a variety of microbial pathogens. The innate and the adaptive immune responses closely collaborate in developing the stage for protective immunity against microorganisms. Early recognition of invading microorganisms is provided by germline-encoded pattern recognition receptors (PRRs) that recognize conserved microbial components known as pathogen-associated molecular patterns (PAMPs). One of the best-characterized PRRs is the still-growing family of Toll-like receptors (TLRs), which are type I integral membrane proteins recognizing such PAMPs as lipopolysaccharide (TLR4), bacterial lipoproteins (TLR2), flagellin (TLR5), and/or CpG DNA (TLR9). The members of this PRRs family are located at cell surface membranes (TLR5, TLR11, TLR4, and the heterodimers of TLR2–TLR1 or TLR2–TLR6), binding to their respective ligands at the cell surface. Others (TLR3, TLR7–TLR8, TLR9, and TLR13) are expressed on endosomal membranes, where they sense microbial and host-derived nucleic acids. TLR4 localizes to both the plasma membrane and the endosomes (see, for example, O'Neill et al., 2013). Ligand-induced dimerization of TLRs leads to signaling by almost all TLRs (except TLR3) using the adaptor protein myeloid differentiation primary response gene 88 (MyD88). MyD88 activates transcription factor NF-κB signaling via serine-threonine kinases IRAK1 and IRAK2 (Warner and Núñez, 2013). For some TLRs, other adaptor proteins are needed to assemble the receptor signaling pathway. Mal (also known as TIR adaptor protein—TIRAP) is necessary to recruit Myd88 to TLR2 and TLR4 to ensure signaling via IRAKs. In the case of TLR4, the MyD88-dependent or MyD88-independent TRIF/TRAM (TIR domain-containing adaptor inducing IFN-β/TRIF-related adaptor molecule) signaling pathways can be activated by lipopolysaccharide (LPS) of Gram-negative bacteria. Ligation of LPS to TLR4 is facilitated by lipopolysaccharide binding protein (LBP) and CD14 (Lu et al., 2008).

**Figure 1:**
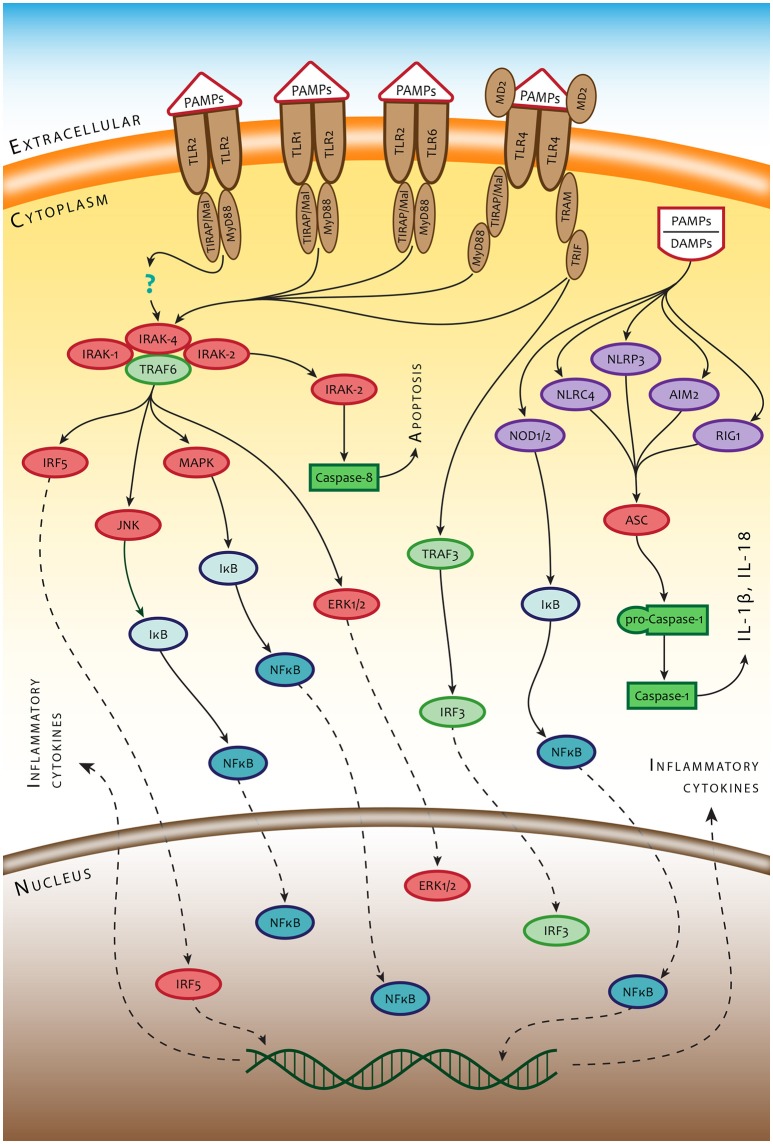
Simplified scheme of innate immune recognition mediated by PRRs. TLR2/TLR1, TLR2/TLR6 heterodimers, and TLR2 homodimer are controlled mainly by the MyD88-dependent signaling pathway and/or the TRIF-dependent signaling pathway using sorting adaptors TIRAP/Mal and TRAM. MyD88 recruits IRAK4, IRAK1, IRAK2 and TRAF6 and induces inflammatory responses by activating NF-κB, MAPK, and IRF5. TRIF recruits TRAF6 and TRAF3, which leads to activation of MAPK and NF-κB. The signals from cell surface PRRs control the ultimate fate of the cell and production of intercellular signals inducing inflammatory response to infection. A different set of PRRs and amplification mechanisms operate in detecting bacteria inside the cytosol. Bacterial small nucleic acids secreted into the cytosol and bacterial mRNA are recognized by RNA-sensing RIG-1 or DNA-sensing Aim2 and NLRP3. Such structural components of bacteria as, for example, flagellin or peptidoglycan are recognized by NLRC4 and NOD1/2 receptors, respectively. Recognition of intracytosolic bacterial nucleic acids activates inflammasome(s) through the adaptor molecule ASC, which leads, in turn, to activation of caspase 1 and production of IL-1 beta (IL-1β) and IL18.

Both macrophages (Mϕ) and dendritic cells (DC), which act as a dominant phagocytic and antigen processing and presentation component of the immune system, are equipped, in addition to TLRs, with numerous membrane-bound and cytosolic receptors that can detect microbes. Among them are complement receptors, C-type lectin receptors (CR), and Fcγ receptors (FcγRs) at the cell membrane, as well as cytosolic nucleotide-binding and oligomerization domain (NOD)-like receptors (NLRs) or interferon-inducible protein, also known as absent in melanoma 2 (AIM2). NLRs and AIM2 constitute the pattern-recognition components of inflammasomes, which sense nucleotide sequences appearing in the cytosol (Kim et al., 2016; Man et al., 2016a). Upon binding a ligand, NLRs as well as AIM2 assemble multiprotein complexes called inflammasomes, which drive pyroptosis and proteolytic cleavage of the proinflammatory cytokines pro-IL-1β and pro-IL-18. The NLRs and/or AIM2 proteins recruit the inflammasome adaptor protein ASC (apoptosis-associated speck-like protein containing a caspase recruitment domain), which in turn interacts with pro-caspase-1 leading to its activation. Once activated, caspase-1 promotes maturation of the proinflammatory cytokines interleukin (IL)-1β and IL-18 (Jin and Xiao, 2015; Xiao, 2015). The general scheme of signaling pathways associated with ligation of PRRs is presented in Figure 1.

Serious infectious diseases in humans and animals caused by intracellular bacteria pose a major concern for the public because, to date, researchers have failed to develop effective vaccines. The reasons lie in the complicated pathogenesis and incomplete understanding of the innate immune recognition processes controlling the generation of immune responses. Knowledge regarding both the innate immune recognition of pathogens and the outfit of pathogens enabling the avoidance of defensive reactions by host cells at the beginning and, subsequently, of the whole host immune system response are the keys to constructing efficient prophylactic tools. Recent evidence supports the concept that the immune response to external stimuli in the form of bacteria is guided by the primary interaction of the bacterium with the host cell. In this review, we provide the basic paradigms of the innate immune recognition concept arising from analyses of data obtained from the various *Francisella* models.

## *Francisella tularensis*—etiological agent of tularemia

*Francisella tularensis (F. tularensis)* is one of the most virulent microorganisms currently known. *Francisellae* are Gram-negative intracellular bacteria causing the zoonotic systemic disease tularemia (Carvalho et al., 2014). Although, the severity of illness varies greatly depending upon which *Francisella* subspecies induces the disease, the taxonomy of the genus *Francisella* is in fact somewhat uncertain. Currently, there are four recognized species: *F. endosymbionts, F. philomiragia, F. novicida*, and *F. tularensis* with three subspecies (*tularensis, holarctica*, and *mediasiatica*; Duncan et al., 2013). The majority of tularemia cases are caused by Type A *F. tularensis* subsp. *tularensis* found exclusively in North America and Type B *F. tularensis* subsp. *holarctica* found throughout the northern hemisphere. A big unknown has long been the taxonomy of *F. novicida*, which is frequently used as a model microorganism to study the pathogenesis of *Francisella* infections. Based on genomic, virulence, pathogenic, clinical, and finally ecological differences, between *F. novicida* and *F. tularensis*, it was recently suggested that *F. novicida* and *F. tularensis* be maintained as separate species (Kingry and Petersen, 2014).

The morbidity and mortality of infection caused by different *F. tularensis* strains vary also according to the gateway of infection. The most dangerous is the pneumonic form of tularemia, followed by gastrointestinal, ulceroglandular, and oculoglandular forms. *Francisella* invades and replicates within phagocytic cell types, such as Mϕ and DC, as well as structural tissue cells, included hepatocytes, alveolar type II cells, or endothelial cells. For this reason, *F. tularensis* has been occasionally called a promiscuous intracellular pathogen (Hall et al., 2008). *F. tularensis* has been previously shown to infect and replicate in Mϕ, both *in vitro* and *in vivo* (Thorpe and Marcus, 1964a,b; Nutter and Myrvik, 1966; Fortier et al., 1994). The attenuated *F. tularensis* type B live vaccine strain (LVS) replicates exponentially in mouse and human DC (Bosio and Dow, 2005; Ben Nasr et al., 2006), and the strain *F. tularensis* Type A Schu S4 efficiently infects and replicates in human myeloid DC (Chase et al., 2009). The CD11b(high) Mϕ, DC, monocytes, and alveolar type II cells in murine lung were shown to be infected after intranasal infection with several strains of *F. tularensis* (Hall et al., 2008). Murine peritoneal Mϕ (F4/80^+^), neutrophils (Gr-1^+^CD11b^+^), and surprisingly almost all B1a B cells (CD19^+^CD5^+^CD11b^+^) have also been shown to be infected at different frequency after experimental intraperitoneal infection induced by LVS (Plzakova et al., 2014).

The prophylaxis of tularemia infection is still problematic. The only vaccine, the live vaccine strain (LVS), is not authorized for human use. The current effort to construct a new *F. tularensis* vaccine is focused on developing both live attenuated and subunit vaccines. Live attenuated vaccine candidates are constructed by deleting genes involved mainly in metabolic and/or virulence pathways, which genes are necessary for *F. tularensis* intracellular replication and *in vivo* survival (Marohn and Barry, 2013). Subunit vaccine construction is oriented to *Francisella* molecular components that induce some degree of protection against lethal respiratory changes, for example surface proteins or lipoproteins administered with appropriate adjuvants or incorporated into liposomes (Putzova et al., 2016). A substantial challenge for vaccine development is to ascertain why *Francisella* seems to be immunologically silent for several days post infection. Vitally needed, therefore, is knowledge regarding host–pathogen interaction in general, and particularly during early stages of the innate immune response that modulate the induction, regulation, and expression of the adaptive immune response.

## Recognition at the host cell membrane

As a Gram-negative bacterium, *F. tularensis* has LPS as a dominant component of its cellular surface. Similar to those of other Gram-negative bacteria, *F. tularensis* LPS is composed of lipid A, which anchors the LPS to the outer membrane, a core oligosaccharide attached to lipid A, 3-deoxy-D-*manno*-octulosonic acid (Kdo), and an O-polysaccharide (also known as O-antigen) which contains a varying number of tetrasaccharide repeating units (Gunn and Ernst, 2007). *Francisella* LPS also has many unusual characteristics, however, and these lead to unexpected consequences during innate immune recognition (Okan and Kasper, 2013). The LPS of gram-negative bacteria is generally recognized by TLR4/MD2, the PRR at the surface of a host cell, and induces a strong proinflammatory response (Maeshima and Fernandez, 2013; Park and Lee, 2013). One therefore could assume that TLR4 will be the dominant PRR recognizing *F. tularensis* at the cell membrane. Purified *F. tularensis* LPS has been shown, however, not to have an agonistic or antagonistic effect on the *Escherichia coli* LPS-induced activation of J774 cells and to have relatively weak endotoxic activity (Sandström et al., 1992; Ancuta et al., 1996; Telepnev et al., 2003; Hajjar et al., 2006). This has been somewhat surprising, because in a previous study the authors reported that TLR4-defective mice (C3H/HeJ strain) were more susceptible than wild-type mice to intradermal infection with LVS (Macela et al., 1996). The limited ability of *F. tularensis* LPS to signal via TLR4 might depend on some structural properties of the lipid A moiety, most likely related to the number and length of the acyl chain substituents and absence of phosphate moieties (Dueñas et al., 2006; Maeshima and Fernandez, 2013). In parallel, it was demonstrated that TLR4 does not contribute to resistance of mice to airborne type A *F. tularensi*s infection or intradermal infection caused by LVS (Chen et al., 2004, 2005). Other studies also have demonstrated the inability of *F. tularensis* LPS to act as either TLR agonists or antagonists (Ancuta et al., 1996; Hajjar et al., 2006). Nevertheless, some constituents of the bacterial body alone can function as TLR4 agonists. For example, the recombinant *F. tularensis* heat shock protein DnaK induced maturation of murine bone marrow-derived DC (demonstrated by an up-regulation of costimulatory molecules CD40, CD80, and CD86) and activated the production of proinflammatory cytokines (IL-6, TNF-alpha, and IL-12 p40, as well as low levels of IL-10) in a TLR4-dependent manner (Ashtekar et al., 2008). These finding may explain the observation (Macela et al., 1996) that TLR4-defective mice are more susceptible than wild-type mice to intradermal infection with LVS. Thus, TLR4 might, to some extent, be engaged in *Francisella* recognition at the cell membrane and, as a coreceptor, could modulate TLR2 signaling pathways downstream.

During the first decade of the twenty-first century, there appeared increasing evidence suggesting that TLR2 is involved in the recognition of *F. tularensis* on the surface of mouse innate immune system cells. TLR2 after ligation recognizes lipid-containing PAMPs such as lipoteichoic acid and di- and tri-acylated cysteine-containing lipopeptides, lipoarabinomannan from mycobacteria, or zymosan from yeast. The specific recognition of ligands by TLR2 is realized either in the form of TLR2 homodimer or as a heterodimer with TLR1 (recognizes tri-acylated lipopeptides) or TLR6 (recognizes the di-acylated ligand; Botos et al., 2011). Within *Francisella* models, TLR2–/– mice had impaired bacterial clearance from livers, lungs, and spleens after intranasal challenge with a sublethal dose of *F. tularensis* LVS. Moreover, infected TLR2–/– mice succumbed to a 10-fold lower challenge dose than did wild-type mice (Malik et al., 2006). Further studies documented that TLR2 of the mouse Mϕ and DC plays a significant role in the recognition of *F. tularensis* and functional activation of their antigen presenting function and controls the proinflammatory cytokine gene transcription (Katz et al., 2006; Li et al., 2006; Malik et al., 2006; Cole et al., 2007). DC from TLR2-deficient mice failed to produce IL-12p70 and did not co-stimulate liver lymphocytes for IFN−γ production in response to viable *F. tularensis* organisms (Hong et al., 2007). TLR2-dependent signaling appears to some extent to control *F. tularensis* infection and modulate inflammatory responses monitored by expression of proinflammatory cytokines and chemokines, such as TNF-α, IL-1β, IL-6, Mϕ inflammatory protein 1α, and Mϕ inflammatory protein 2 (Katz et al., 2006; Li et al., 2006; Malik et al., 2006; Abplanalp et al., 2009). TLR2 signaling seems to be dependent on new bacterial protein synthesis because the TLR2 agonist activity was abrogated when *F. tularensis* LVS organisms were heat- or formalin-killed or treated with chloramphenicol (Cole et al., 2007).

A substantial number of studies have identified TLR2 signaling as a critical event during the host innate immune response to *F. tularensis* infection. The data may indicate that, in a *Francisella* model, the TLR2 alone (perhaps as a homodimer—see for example Zheng et al., 2015; Udgata et al., 2016), rather than TLR2/TLR1 or TLR2/TLR6 heterodimers, play a critical role in *F. tularensis* recognition at the surface of immunocompetent cells (Abplanalp et al., 2009). Furthermore, a TLR2-independent pathway for activation of macrophages has also been identified (Hong et al., 2007). Contrary to the majority of information, some data suggest that signals utilizing the adaptor protein MyD88 without the involvement of TLR2 are essential for controlling resistance to intradermal challenge with *F. tularensis* LVS but not for intra-macrophage bacterial multiplication (Collazo et al., 2006). Adaptor protein MyD88 is used by almost all TLRs (except TLR3) to activate the transcription factor NF-κB (Lord et al., 1990). TIRAP/Mal (TIR domain-containing adaptor protein/MyD88-adaptor-like), an adaptor protein closely related to MyD88, is necessary to recruit Myd88 to TLR2 as well as to TLR4 (Horng et al., 2001, 2002). In the context of other *Francisella* studies it was demonstrated that the molecular complex of TLR2/MyD88 (signaling through IRAKs; Arancibia et al., 2007) is indispensable for NF-κB activation initiating macrophage proinflammatory cytokine production and, subsequently, protective innate immune responses in mice following challenge with attenuated as well as virulent *F. tularensis* strains (Collazo et al., 2006; Abplanalp et al., 2009; Cole et al., 2010; Russo et al., 2013).

Recognition of *Francisellae* at the host cell membrane is a fundamental step in its life cycle because it facilitates bacterial entry into host cells. This event is not exclusively a matter of TLRs. Such receptors as C-type lectin receptors, complement receptors (CR), and Fc-gamma receptors (FcγR) were shown in a specific situation to recognize either unopsonized or opsonized bacteria. The presence of natural opsonins in an experimental setup has a major role in the early phases of host–pathogen interactions and alters the intracellular fate of bacteria (Dai et al., 2013). Several models of *Francisella*–host cell interaction have identified the receptors engaged in the *Francisella* uptake at the host cells surface. The mannose receptor, one of the C-type lectin receptors (Balagopal et al., 2006; Schulert and Allen, 2006), the complement receptors CR3 (CD11b/CD18) in the case of Mϕ (Clemens et al., 2005; Balagopal et al., 2006; Geier and Celli, 2011), CR4 (CD11c/CD18) in the case of DC (Ben Nasr et al., 2006), and CR1/2 in the case of B cells (Plzakova et al., 2015), the scavenger receptor A (SRA) (Pierini, 2006; Geier and Celli, 2011), FcγRs (Balagopal et al., 2006; Geier and Celli, 2011), and surface-exposed nucleolin with its bacterial ligand EF-Tu (Barel et al., 2008, 2010; Barel and Charbit, 2014) are involved in internalization of *Francisellae* into host cells.

Opsonization of *F. tularensis* Schu S4 strain with fresh serum or purified antibodies reoriented the interaction of bacteria with mouse bone marrow-derived Mϕ from the mannose receptor to the complement receptor CR3, the scavenger receptor A (SRA), and the FcγR (Geier and Celli, 2011). Experimental data demonstrated that opsonization of bacteria prior to engulfment by phagocytes substantially changes the intracellular fate of the bacteria and modulates parameters of the host APCs response to infection. CR3-mediated uptake of *Francisellae* negatively modulated maturation of the early *Francisella*-containing phagosome (FCP) and minimize phagosomal escape, whereas FcγR-dependent phagocytosis was associated with intensive superoxide production in the early FCP, a rapid, FcγR-mediated, NADPH oxidase-dependent oxidative burst, and restricted phagosomal escape (Geier and Celli, 2011). Serum opsonins modulate maturation of human monocyte-derived immature DC and change their cytokine production profile in favor of IL-10 at the expense of IL-12 production (Ben Nasr et al., 2006). Efficient attachment and uptake of the highly virulent Type A *F. tularensis* subsp. *tularensis* strain Schu S4 by human monocyte-derived macrophages (hMDMs) require complement C3 opsonization and CR3. A complex cascade of events ending in uptake of *Francisellae* by phagocytes can initiate natural IgM binding to surface capsular and/or O-Ag polysaccharides of *F. tularensis*, a process activating classical complement cascade via C1q and promoting C3 opsonization of the bacterium and phagocytosis via CRs in a phagocyte-specific manner. CR1 (CD35) and CR3 (CD11b/CD18) have been observed to act in concert for phagocytosis of opsonized *F. tularensis* by human neutrophils, whereas CR3 and CR4 (CD11c/CD18) mediated uptake by hMDMs (Schwartz et al., 2012b). However, the CR3 engagement in an efficient uptake of *Francisellae* by hMDMs, in parallel, initiated CR3–TLR2 crosstalk leading to down-regulation of TLR2-dependent proinflammatory responses by inhibiting MAPK activation through outside–in signaling (Dai et al., 2013). Thus, such complex activation of several receptor signaling pathways influences the result of host–microbe interaction.

Taken together, the entry of *Francisellae* into host cells can be realized either by uptake of unopsonized or opsonized bacteria. In real *in vivo* situations, uring natural infections, however, the uptake of opsonized bacteria is probably much more frequent than contact of host cells with unopsonized microbes. This latter mode of cell infection comes into play only during phagocytosis of whole infected cells by bystander phagocytes or during cytosolic transfer between macrophages via the process known as trogocytosis (Bourdonnay and Henry, 2016; Steele et al., 2016). Engagement of opsonophagocytic receptors alters the intracellular trafficking of *Francisella* by modulating the phagocytic pathways that restrict phagosomal escape and intracellular proliferation. Both can impact profoundly the final fate of *Francisella* in a host by modulating the intracellular recognition of *Francisella* in a cytosolic compartment of a host cell. These facts should be taken into account when designing *in vitro* experimental cell infection systems.

## Innate recognition at intracellular compartments

Uptake of *Francisella* by host cells is dependent on actin polymerization and functional microtubules in both phagocytic and nonphagocytic cells (Clemens and Horwitz, 2007; Lindemann et al., 2007). The mechanism of *Francisella* entry into host cells is dependent on the host cell type and the conditions under which the interaction with host cells takes place. One of the specific forms of entry is the formation of asymmetric, spacious pseudopod loops around live or killed bacteria (Clemens et al., 2005). More general mechanisms include macropinocytosis that had been demonstrated for the entry of *Francisella* LVS into type II alveolar epithelial cells or, rarely, during infection of macrophages with LVS (Clemens et al., 2005; Bradburne et al., 2013). Early after interaction with host cell surface receptors *Francisella* is enclosed in a phagosome. There is no information available as to whether the bacteria are recognized during this stage of intracellular trafficking in spite of the fact that phagosomal PRRs recognize bacterial molecular markers (for example, phagosomal TLR9 has the capacity to recognize bacterial CpG motifs). The only information on intracellular colocalization of *Francisella* and TLR2 and MyD88 within macrophages suggests that *Francisella* LVS initiates signaling through TLR2 both at the cell surface and within the phagosome (Cole et al., 2007). The original engagement of the cell membrane PRRs and opsonophagocytic receptors on the cell surface, through which the mutual interaction of *Francisella* with a host cell is realized, produces the signals that dictate the fate of *Francisella* during intracellular trafficking (see, for example, Geier and Celli, 2011). Through modulation of phagosome biogenesis, *Francisella* escapes from its initial phagosome into the cytosol of a host macrophage.

Escape from the phagosome is the second fundamental step in the *Francisella* life cycle. This event is very dynamic, and experimental data has documented that phagosomal escape occurs within several tens of minutes to several hours post infection, depending upon the experimental setup (Golovliov et al., 2003; Clemens et al., 2004; Santic et al., 2005, 2008; Checroun et al., 2006). The cytosolic compartment of infected cells enables the proliferation of *Francisellae*. Once in the cytosol, however, the bacterial load is monitored by intracellular cytosolic DNA sensors, such as DNA-dependent activator of IFN-regulatory sensor (Takaoka et al., 2007), RIG-I (Ablasser et al., 2009), and/or AIM2 (Fernandes-Alnemri et al., 2009; Hornung et al., 2009; Jones et al., 2010), or by cytosolic nuclear oligomerization domain (NOD)-like receptors (NLRs) (Franchi et al., 2008, 2009). Both types of sensors are critical for innate defense by recognizing conserved structures of microorganisms. Upon sensing adequate ligands, these cytosolic PRRs trigger oligomerization of the inflammasome complex. Once completed, inflammasomes interact with 45-kDa pro-caspase 1, which undergoes auto-proteolytic processing that results in active caspase 1 (Thornberry et al., 1992; Miller et al., 1993; Ayala et al., 1994; Wilson et al., 1994). Subsequent cleavage of pro-IL-1β and pro-IL-18 into their mature forms is critical for the host response to infection and is accompanied by caspase-1-dependent inflammatory cell death—pyroptosis (Man and Kanneganti, 2015).

Most information related to cytosolic recognition of *Francisella* has originated from *F. novicida* experimental models. Mice lacking AIM2, ASC, or caspase-1 are highly susceptible to infection and exhibit an increased bacterial burden compared with wild-type mice (Mariathasan et al., 2005; Fernandes-Alnemri et al., 2010; Jones et al., 2010). Macrophages from mice lacking AIM2 cannot sense cytosolic double-stranded DNA and fail to trigger inflammasome assembly. Immunofluorescence microscopy of macrophages infected with *Francisella* further revealed striking colocalization of bacterial DNA with endogenous AIM2 and inflammasome adaptor ASC (Jones et al., 2010). For the intracytosolic recognition of *Francisella*, therefore, the assembly of inflammasome and the accessibility of bacterial DNA or other molecular components of the bacteria are key events. In the case of AIM2 inflammasome and *Francisella*, the critical role seems to be the integration of innate immune signaling. TLR2 signaling through MyD88 and NF-κB in macrophages infected with *F. novicida* contributes to the rapid induction of inflammasome assembly and inflammasome functional activation (Jones and Weiss, 2011). How the recognizable bacterial components in the cytosol are produced is still under investigation. They can be produced directly in the cytosol or, alternatively, can originate from dead, partially destroyed bacteria released into the cytosol after fragmentation of the phagosomal membrane by live, fully active bacterial partners entrapped in a phagosome together with dead ones. For direct destruction of bacteria residing in the cytosol it can be argued that assembly and activation of the AIM2 inflammasome during infection with *F. novicida* requires transcription factor IRF1. The DNA sensor cGAS and its adaptor STING (Sun et al., 2013; Wu et al., 2013) induce type I interferon-dependent expression of IRF1. The IRF1 subsequently modulates the expression of guanylate-binding proteins (GBPs) that can ensure intracellular killing of bacteria and mediate cytosolic release of ligands for recognition by the AIM2 inflammasome (Man et al., 2015). Moreover, the interferon-inducible protein IRGB10 participates in the cytosolic destructive process of *F. novicida* by a mechanism requiring guanylate-binding proteins (Man et al., 2016b). Just because they are recognized by cytosolic DNA sensors does not necessarily mean that the bacteria will be killed; their product, cyclic dinucleotides, is sensed by STING directly and can initiate the innate immune response (see below).

Evidence of type I IFN involvement in the process of cytosolic recognition of *Francisella* by the inflammasome documents the need for multifold signal integration during adaptation of cells to recognize intracellular pathogens during primary interaction with host cells (Henry et al., 2007). Type I IFN signaling was observed to be necessary for activation of the inflammasome during infection with *F. novicida*. Production of type I IFN was coupled with recognition of cytosolic *F. novicida*. The process of *F. novicida* recognition was dependent on IRF-3 signaling and independent of signaling from RIG-I, MDA5, Nod1/2, and inflammasome adaptors. It was also independent of TLR signaling, which evidently demonstrated the intracytosolic recognition process (Henry et al., 2007). Production of type I IFN by BMDM after infection with *F. tularensis* LVS was observed to be dependent on STING, also known as MITA, MPYS, ERIS, and TMEM173. STING functions as both a direct cytosolic DNA sensor and an adaptor protein utilizing different molecular mechanisms (Ishikawa and Barber, 2008; Ishikawa et al., 2009). Downstream, STING activate transcription factors STAT6 and IRF3 through kinase TBK1 (Burdette and Vance, 2013). Signaling utilizing STING by cultured macrophages infected with LVS was required for type I IFN production, however, in parallel; a STING-dependent as well as a STING-independent signaling pathway were activated in *in vivo Francisella* infection models (Jin et al., 2011). The cyclic GMP-AMP synthase cGAS (Sun et al., 2013), as well as IFI214, a murine homolog of IFI16 (Unterholzner et al., 2010; Veeranki and Choubey, 2012), act as cytosolic DNA sensors and seem to be involved in the sensing of intracytosolic *Francisella* DNA; both contribute to STING-dependent type I IFN response to high concentrations of cytosolic dsDNA (Storek et al., 2015). Moreover, as a direct innate immune sensor, STING alone can recognize cyclic dinucleotides (c-di-AMP and c-di-GMP), which are produced by bacteria, and mediate type I IFN cell response (Burdette et al., 2011; Barker et al., 2013).

To recapitulate and summarize the studies on intracytosolic recognition of *Francisella*, there are multiple intracytosolic signaling pathways that can, alone or in tandem, ensure the recognition of *Francisellae* localized in cytosolic compartment of a host cell. Reasons for these findings may be several. Unterholzner (2013) summarizes possible answers to the question of why so many receptors recognize DNA localized in the cytosol (nucleus) and induce an interferon response. First, receptors may have redundant functions. Further, DNA receptors may differ in their ligand specificity, different DNA receptors operate in different cell types, and/or receptors may act sequentially over time. Finally, some of the proposed DNA sensors may not be receptors. For our model of the intracellular pathogen *Francisella*, it can be concluded that at least two basic intracytosolic recognition processes are indispensable for expression of a proinflammatory response to infection. First, and in general, it is such recognition which results in the production of type I IFN needed for activation of IRFs. The second process is the expression of components and their assembly into inflammasome, by which the *Francisella* is recognized and the production of proinflammatory IL-1β and IL-18 cytokines is ensured.

## Signaling windows concept—spatiotemporal network of cellular hosts

The emerging concept of signaling windows of innate immune recognition is based on the idea that there exist functional cellular immune response modules that temporarily, in spatiotemporal configuration, regulate innate immune recognition and sequentially modulate induction, regulation, and expression of the adaptive immune response. Intracellular pathogens such as *Francisella* are ideal models to construct realistic scenarios of innate immune recognition. Utilizing integrated approaches and analyzing the fate of mutual host cell–pathogen interaction, recent complex dynamic studies on immune cell networking have attempted to overcome the traditional static view on induction of immune cell signaling during induction of the immune response (see, for example, Nunes-Alves et al., 2014; Budak et al., 2015; Hotson et al., 2016; Rothchild et al., 2016). The *Francisella* experimental infection models provide sufficient information for attempting to construct the architecture of innate immune signaling windows. The basic paradigms of the signaling window concept define, in the strict sense, a cell as a decisive host of microbes and accept the idea of functional cellular modules of immune responses. The concept encompasses several basic assumptions: (1) There is a “jumble” of cellular hosts within a multicellular organism infected by bacteria. (2) Cellular hosts create a four-dimensional net in the host organism. (3) Cellular hosts recognize and respond to interaction with the bacterium on spatiotemporal levels. (4) The host cell with which a microbe originally interacts forms the first signaling window. (5) Every spatiotemporal level opens a new signaling window. (6) The interplay among individual signaling windows ensures paracrine cytokine messages induced by microbial challenge. (7) Signaling windows integrate innate immune recognition signals. (8) PRRs cross-inhibition and interference of host and microbe signals influence the result of host–microbe interaction.

Macrophages and DC, which have been most utilized for studies on innate immune recognition, are not the only cells that recognize *Francisellae* in the context of the innate immune response. Moreover, TLRs are not the only cell surface receptors engaged in the innate immune recognition. *Francisella* infects phagocytic as well as nonphagocytic cells. Along with macrophages and DC, neutrophils (Löfgren et al., 1983; Hall et al., 2008), B cells (Plzakova et al., 2014), endothelial cells (Forestal et al., 2003), epithelial cells (Gentry et al., 2007; Lindemann et al., 2007), and/or hepatocytes (Law et al., 2011; Rennert et al., 2016) are all potential primary targets of *Francisella*. All these are permissive, and all are significant producers of signals representing the first signaling window. Neutrophils utilize a combination of NADPH oxidase-derived reactive oxygen species (ROS), antimicrobial peptides, and degradative enzymes to kill engulfed microorganisms for innate host defense (Kennedy and DeLeo, 2009). Phagocytosis of microorganisms leads very quickly to neutrophil apoptosis, which is known also as phagocytosis-induced cell death (Kobayashi et al., 2003). Tested strains of *Francisella*, however, inhibit the respiratory burst and profoundly prolong neutrophil lifespan (Schwartz et al., 2012a). In parallel, at the gene level, *Francisella* infection of neutrophils enhances expression of such neutrophil-specific survival factors as *cdk2* or *cdk7*, cytokine and chemokine genes that promote inflammation as well as neutrophil survival (*Il1b, Il1rn, Il6, osm, pbef1, cxcl1, 0ccl4, cxcr4*). *Francisella* also has been shown to significantly affect expression of genes associated with cytosolic pattern recognition systems and inflammasome activation, as well as with early induction of *NLRP3* and *NOD2* followed by down-regulation of *AIM2, NAIP, PYCARDI*, and *NLRP1*. As in other cells that may be regarded as providing the cellular background of the first signaling window, *Francisella* escapes from the phagosome into the neutrophil cytosol (McCaffrey and Allen, 2006), where it might be recognized by inflammasomes or NOD-like receptors. B cells are engaged in the strong early protective response against *F. tularensis* LVS (Culkin et al., 1997). As early as 12 h post infection, peritoneal CD19(+) cells produce IFN-γ, IL-1β, IL-4, IL-6, IL-12, IL-17, IL-23, and TNF-α (Plzakova et al., 2014). Mice deficient in mature B cells and antibodies (B-cell knockout mice) actually control primary sublethal infection but are 100-fold less well protected against a secondary lethal challenge (Elkins et al., 1999). Direct contact and entry of *Francisella* into B cells, depending on a given B cell subset, are mediated by B cell receptors (BCRs) with or without complement receptor CR1/2. In the B-1a cell subset, BCRs alone can ensure the internalization process, whereas BCRs on B-1b and B-2 cells require co-signaling from the coreceptor containing CR1/2 in order to initiate *F. tularensis* engulfment (Plzakova et al., 2015). The production of IL-1β by infected B cells suggests early cytosolic innate immune recognition after interaction of *Francisella* and B cell. At some stage of dissemination into various organs, *Francisella* must overcome the endothelial barrier in the microvasculature by one of three well-known mechanisms: transcellular, paracellular, or the so-called Trojan horse mechanism (i.e., crossing the barrier using infected phagocytes). To achieve this, *Francisella* readily adheres to the endothelial cell surface and uses PilE4 (type IV pili subunit) to interact with ICAM-1 molecule, adhere to the endothelial surface, and cross the endothelial barrier *in vivo* as well as *in vitro* (Bencurova et al., 2015). Endothelial as well as epithelial cells and hepatocytes produce a variety of cytokines at various levels after interaction with bacteria. This is just a reason for modulation of the functional profile of cells subsequently interacting with *Francisellae*, and in the suggested concept it corresponds to opening of the secondary signaling windows.

As part of innate immune response, moreover, unconventional T cell subsets might be engaged in innate immune recognition and constitute a bridge between innate and adaptive immune responses. This might be achieved through unconventionally presented byproducts or intermediates of bacterial metabolism secreted by the bacteria early after interaction with the host cells. Human Vγ9/Vδ2 T cells recognize specifically, in a non-MHC restricted manner, microbial isoprenoid precursor (*E*)-4-hydroxy-3-methyl-but-2-enyl pyrophosphate (HMB-PP), which is an intermediate of the microbial non-mevalonate pathway of isoprenoid biosynthesis utilized by most pathogenic Gram-negative and Gram-positive bacteria (Eberl and Jomaa, 2003; Eberl et al., 2003; Heuston et al., 2012). This molecule can function as a danger signal because HMB-PP is not present in higher eukaryotes, including humans (Sicard and Fournie, 2000; Morita et al., 2007). One of the consequences of the human Vγ9/Vδ2 T cells–pathogen interaction, and, in a broader sense, of their activation, is the rapid acquisition of antigen presenting cell characteristics that are reminiscent of mature DC (Moser and Eberl, 2011; Tyler et al., 2015), and in such way they can function as a primary signaling window. Concerning human tularemia, Vγ9/Vδ2 T cells expand after infection and comprise on average 30% of human peripheral blood T lymphocytes, thus suggesting some role in control of *F. tularensis* infection (Poquet et al., 1998). Experiments with co-culture of human Vγ9/Vδ2 T cells isolated from healthy donors with the THP-1 human monocyte cell line infected with *F. tularensis* has demonstrated the ability of Vγ9/Vδ2 T cells to recognize infection, to produce a whole range of cytokines and chemokines (including IL-1β, IL-6, and IFN-γ, but not IL-10), and to limit bacterial proliferation in the culture (Rowland et al., 2012). Another unconventional T cell subset, mucosa-associated invariant T (MAIT) cells, also responds very early and intensively to *F. tularensis* infection. A murine *in vivo* model of sublethal *F. tularensis* LVS pulmonary infection demonstrated robust expansion of MAIT cells in the lungs during the early acute phase of infection. The MAIT cells recognized vitamin B metabolites in association with evolutionarily conserved MHC-related protein 1 (Kjer-Nielsen et al., 2012), which possesses a unique antigen-binding cleft producing an antigen presenting function on this cell type (Huang et al., 2005). Because vitamin B biosynthesis pathways are unique to bacteria and yeast, MAIT cells may also constitute one of the primary signaling windows.

**Figure 2:**
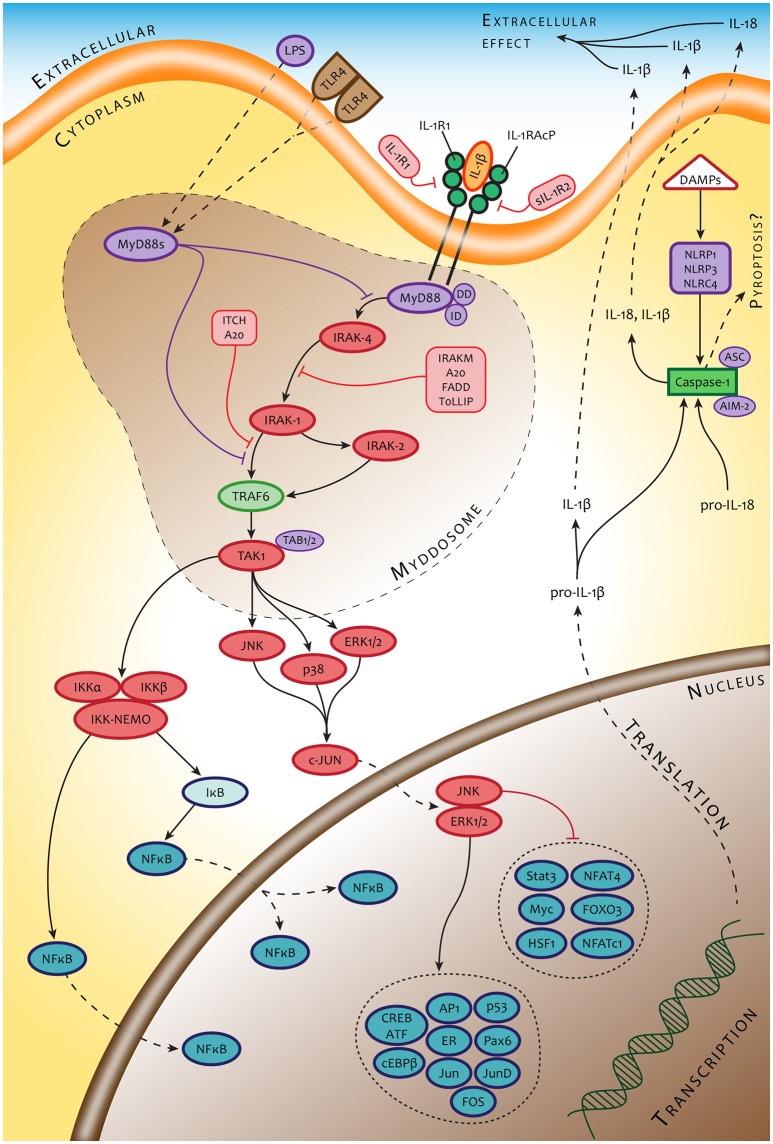
An example of signaling pathways of cells interacting with *Francisella* in secondary order. The signaling pathways of these cells are modulated by signals originating from a primary responding cell (IL-1β) or collateral signals from invading microbes (LPS).

Knowing that the severity and time course of tularemia is, at least in an experimental setup, dependent on the route of infection, we can accept the view that these differences are due, at least in part, to the various types of original cellular hosts that convey primary interaction with the bacteria. Thus, the initial signals originating from different infected cell types might dictate the outcome of primary host cell–bacterium interaction, which in turn affects all subsequent events during the induction of immune responses. The cells that interact with the *Francisellae* in the first sequence create a timeframe for modulating the functional profile of cells that will interact with the bacterium in a secondary order. The intercellular contact is mediated by an integrated cytokine message produced by originally infected cellular “senders.” To our knowledge, the lifetime of infected cells in the case of macrophages is from 12 to 18 h post infection, depending on the experimental model (Libich, 1981). The transient bacteremia contributing to spreading *Francisellae* over the body begins 24–48 h post infection, which is thus the timeframe for changing the microenvironment for as yet uninfected cells. Cellular “receivers” located in a given microenvironment can thus respond accordingly. We hypothesize that the modulated functional profile of a secondarily reacting cell will be dependent on the type of cell that initiates the cytokine messages as well as on the expressed cell surface receptors able to recognize the cytokine messages and simultaneously (or subsequently) recognize incoming bacterium. The cytokine response of a cellular “receiver” of the same cell type as the cellular “sender,” whether infected or not, would necessarily be different from the message produced by the originally infected cell. Moreover, in the case of antigen presenting cells reacting in the secondary order, the recognition, handling, and processing the interacting bacteria might be quite different from the processes of the primary infected cells and could have a profound impact on the expression of the adaptive immune response. An example is the paracrine action of type I IFN, which changes the transcriptional response to innate immune recognition of *Francisellae* by infected primary human monocyte-derived macrophages and primary murine peritoneal macrophages but not by murine bone marrow-derived macrophages. This type I IFN-dependent modulated response of infected cells is synergistic with TLR2 transcriptional responses, partially TLR2-independent, but strictly MyD88-dependent, thus suggesting the supplementary action of co-receptor(s) (Richard et al., 2016). Alternatively, it could demonstrate the modulated function of cells reacting to *Francisellae* as a receiver of signals generated by cells infected in the primary order. An example can be seen in the signaling through IL-1 receptor of cells infected with *Francisellae* in a secondary order (Figure 2).

Thus, in our opinion, the data from the literature indicate that the differences in severity of tularemia depending on the route of infection can originate from differences in the local microenvironments and types of cells that are first infected with *Francisella*. As an example, the difference in LD50 after aerogenic and subcutaneous *F. tularensis* infection of mice is greater than six logarithms. Route of infection dominates over genetic background of recipients in the severity of illness. In the case of genetic background, the difference in LD50 for susceptible and resistant mice is less than three logarithms (Fortier et al., 1994). The reason for these differences can be seen in the different primary cellular hosts of *Francisellae*. Langerhans cells are primarily infected in dermis whereas alveolar macrophages are predominantly infected in the lungs. Such conclusion can also be deduced by comparing intranasal- vs. intradermal-induced murine tularemia (Roberts et al., 2014), which may be an example testifying to the concept proposed above.

The basic experiments with individual cell types and opsonizing bacteria have demonstrated not only the importance of responding cell type to primary interaction with *Francisella* but also the importance of type of expressed surface membrane receptor involved in the primary contact with the bacterium. Complement factors, such as ubiquitous opsonins in mammals, are critical factors mediating adhesion and subsequently internalization of *Francisellae* into host cells. The co-signaling from different surface receptors engaged in the adhesion of the bacterium modulates the response of host cells. There is an obvious dichotomy in macrophage response to interaction with C3 opsonized or unopsonized microbes, especially in relation to inflammatory response due to the interference of signaling pathways (Dai et al., 2013). Moreover, *Francisella* itself modulates the signaling pathways of infected cells. The data demonstrate, for example, that *F. tularensis* fails to induce production of proinflammatory cytokines or IFN-γ, inhibits increased expression of activation markers, including MHCII, on the surface of professional APCs, and suppresses activation of the inflammasome during early *Francisella* infection via targeting of TLR2-dependent signaling (Bosio and Dow, 2005; Bosio et al., 2007; Parsa et al., 2008; Chase et al., 2009; Dotson et al., 2013). There is uncertainty in the interpretation of these results, however, because much of this data was generated using different experimental setups and in different time proportions. Moreover, the majority of these studies were based on entirely artificial conditions not corresponding to *in vivo* situations (natural opsonization of bacteria and very high multiplicity, which is quite exceptional for natural infection). Some conclusions have been generalized according to the final fate of the eukaryotic host cell/organism–pathogen interaction using a traditional, more or less static experimental arrangement. To solve the general problem of innate immune recognition's involvement in the process of protective immunity induction, analyses are needed of the so-called social network of immune cells that participate in the early stages of infection.

To conclude the possible sequence of events during innate immune recognition of *Francisella* according to the emerging concept of signaling windows, we can formulate a minimum of four basic assumptions: (1) The first batch of signals, resulting from natural recognition of *Francisella* inside the multicellular organism, depends on the type of interacting cell and its surface receptor(s), which mediate(s) primary pathogen conjunction with the host cell. (2) *Francisella*–host cell interaction at the single-cell level corresponds to the concept of crosstalk between innate immune receptors and integrates the signals into a prototypic signaling response corresponding to a particular cell type. (3) During the process of *Francisella* innate immune recognition, cells form a four-dimensional signaling network represented by signaling windows. This means that the concept reflects the spatiotemporal changes in the function of the individual cells that are engaged in immune recognition, which are caused by changing microenvironment over time. Integrated signals at the level of signaling windows generate a new signal for “opening” the additional signaling window. (4) Interference of host and microbe signals at a single-cell level can subvert and reprogram PPRs-mediated innate immune responses.

## Conclusions and prospects

Innate immune response constitutes the first line of defense against bacterial infection. Data collected from germ-free as well as specific pathogen free animal models of microbial pathogenesis demonstrate the dependency of induce and adaptive immunity on primary interaction between a microorganism and the host cell that the microbe first encounters. The innate immune recognition process plays a dominant role along with the intrinsic characteristics of the microorganism and host. The epigenetic reprogramming of innate immune cells, creating the hierarchy of immune response functional modules, is critical for inducing and regulating expression of the adaptive immune response. This process is extremely dynamic and the populations of individual cell types, whether infected or uninfected, change their functional characteristics depending on the local microenvironment modulated by the cells that were originally infected even at a distant place. If this is the case, then not all the cells of the same cell type in the body will respond to infection in an identical way at a given time. From this point of view, the cell, with its functional and secretion profile, rather than the host organism in its entirety, seems to be the primary microbe host (Kubelkova et al., 2016). In the case of *in vitro Francisella* models, the literature presents quite different conclusions concerning the outcome of *Francisella* innate immune recognition. Some studies provide evidence that *F. tularensis* LVS represses inflammasome activation, while other studies have demonstrated that *F. tularensis* LVS increases mRNA levels of proinflammatory cytokines and this is followed by increased protein secretion. Moreover, data from experiments with host-adapted *Francisella* LVS isolated from infected macrophages explain this heterogeneity of results by a “stealthy mode” of intra-host lifestyle (for a review, see Holland et al., 2016).

The host cells as well as invading *Francisellae* are affected by their “historical memory” and mutually generate, at any given time, an immediate microenvironment that should be respected in the generation of new infection models. In our experience, for example, germ-free mice infected subcutaneously with *F. tularensis* reacted differently to attenuated and virulent strains in comparison with specific-pathogen free mice. These mice, not having had contact with bacteria in ontogeny, can demonstrate the unique primary reaction of their immune systems to a pathogenic bacterium and can help us to understand the processes that lead to the establishment of full-fledged protective immunity. To understand the innate immune response to infection we need to obtain multidimensional data sets providing comprehensive, cell-specific, and time-structured information on epigenetic reprogramming of innate immune cells that can provide us with the logic of interplay among immune cells.

## Author contributions

All authors listed have made a substantial, direct, and intellectual contribution to the work, and approved it for publication.

### Conflict of interest statement

The authors declare that the research was conducted in the absence of any commercial or financial relationships that could be construed as a potential conflict of interest.

## Abbreviations

AIM2: absent in melanoma 2
BCR: B cell receptor
CR: complement receptor
hMDMs: human monocyte-derived macrophages
LPS: lipopolysaccharide
LVS: live vaccine strain
MAIT cells: mucosa-associated invariant T cells
MyD88: myeloid differentiation primary response protein
NLRs: nucleotide-binding and oligomerization domain (NOD)-like receptors
PAMPs: pathogen-associated molecular patterns
PRRs: pattern recognition receptors
TLRs: Toll-like receptors.
